# The Control of Electromagnetic Fields at Work Regulations 2016 and medical MRI

**DOI:** 10.1259/bjr.20160813

**Published:** 2017-02

**Authors:** Stephen F Keevil, David J Lomas

**Affiliations:** ^1^ Department of Medical Physics, St Thomas' Hospital, Guy's and St Thomas' NHS Foundation Trust, London, UK; ^2^ Department of Biomedical Engineering, King's College London, St Thomas' Hospital, London, UK; ^3^ Departments of Radiology, University of Cambridge and Addenbrooke's Hospital, Cambridge, UK

## Abstract

This short commentary provides the MRI community in the UK with practical advice on the impact of the Control of Electromagnetic Fields at Work Regulations 2016 in clinical and research settings. The regulations are the UK implementation of the European Union Physical Agents (Electromagnetic Fields) Directive, which has been the subject of much discussion and concern over the past 13 years. However, thanks to concessions achieved through negotiation, and sensible and proportionate transposition into UK law by the Health and Safety Executive, the negative consequences that were foreseen have been averted. MRI activities are exempt from the occupational exposure limits contained in the regulations, subject to meeting certain conditions. The commentary gives advice on compliance with these conditions and on how to satisfy the other requirements of the regulations, all of which are either already required under existing legislation or represent good MR safety practice.

## COMMENTARY

The Control of Electromagnetic Fields at Work Regulations 2016 (known as the CEMFAW Regulations)^1^ came into force in the UK on 1 July 2016. Equivalent (and for our purposes identical) regulations came into force in Northern Ireland on 1 August 2016.^2^ The purpose of this legislation is to implement the European Union (EU) Physical Agents (Electromagnetic Fields) Directive (2013/35/EU)^3^ in UK law. Members of the MRI community will be very aware of the lengthy campaign that was undertaken to mitigate the impact of this directive and its predecessor^4^ on MRI in clinical practice and research.^5–7^ They may also recall that the 2013 directive includes a derogation (or exemption) for most applications of MRI from the very conservative electromagnetic field (EMF) exposure limits that would have had such a detrimental effect on our sector. However, concerns remained about the vague and ambiguous conditions attached to this derogation and how they would be implemented and interpreted at the national level.^7^ The good news is that the Health and Safety Executive (HSE) worked very closely with the MRI community when the CEMFAW Regulations were being drafted. The regulations are much clearer and better structured than the directive, the conditions attached to the MRI derogation have been rewritten in a more transparent and sensible way and the resulting impact on MRI facilities is expected to be minimal.

The HSE has issued a guide to the regulations,^8^ which provides some useful information in the context of MRI, but is mostly generic and also applies to other employment sectors that are required to comply with the exposure limits, since they are not covered by the derogation. The European Commission has also issued a guide to implementing the directive.^9^ This does discuss the MRI derogation (particularly in Appendix F), but is not specific to the UK context and does not contain the more helpful wording that has been adopted in the CEMFAW Regulations. The aim of this commentary, prepared on behalf of the British Institute of Radiology (BIR) MR Safety Working Party, is to summarize the implications of the regulations in the specific context of clinical and research MRI in the UK and explain what MRI facilities will need to do to comply. This advice is intended to be generic and to be applicable to all UK MRI facilities, irrespective of specific scanner specifications such as the vendor, field strength and gradient performance. The HSE has reviewed a draft of the commentary and has agreed that it reflects their approach in relation to the transposition of the directive. The BIR, together with other relevant professional bodies, is planning to issue more detailed sector-specific national guidance in due course.

One thing that should be made clear, as it has already been a source of misunderstanding, is that the CEMFAW Regulations are UK legislation and so are unaffected by “Brexit”. They will remain in force when the UK leaves the EU, unless and until the Parliament chooses to repeal them.

The regulations generally require employers to ensure that worker exposure to EMFs does not exceed certain exposure limit values (ELVs). However, under Regulation 4(3) (b), this requirement does not apply to “the development, testing, installation, use and maintenance of, or research related to, magnetic resonance imaging equipment for patients in the health sector”. The wording of this exemption is very similar to that contained in the directive itself, which was developed during negotiation in order to capture as much clinical and research MRI activity as possible, including supporting activities such as testing and cleaning. It seems reasonable to assume that this exemption encompasses all activities carried out in an MRI facility that are related to clinical service provision or clinical research, including spectroscopy and other non-imaging procedures carried out using “magnetic resonance imaging equipment”. The wording does appear to exclude pre-clinical and basic science research and certainly does not extend to non-medical applications of MRI or use of MRI in veterinary practice (HSE lawyers were clear that pet animals cannot be regarded as “patients” in this context). However, on the same day that the regulations came into force, the HSE issued a “Certificate of Exemption” under Regulation 13, which allows “other work activities” to be exempted from the ELVs (in exercise of the “discretionary derogation” power contained in the directive). The activities listed in this Certificate of Exemption (available from: http://www.hse.gov.uk/radiation/nonionising/emf-exemption-certificate.pdf) include “the use of magnetic resonance imaging equipment other than for patients in the health sector”. Therefore, in the UK at least, all uses of MRI are exempt from the ELVs contained in the EMF directive, as long as the necessary conditions are satisfied. It is also worth mentioning that these additional discretionary exemptions granted under the HSE Certificate of Exemption are in place for 5 years in the first instance, as the directive requires them to be kept under review. However, there is nothing to prevent exemptions being renewed at that point.

The conditions attached to the derogation were one of the main remaining concerns with the 2013 directive. However, the HSE has gone as far as possible in simplifying and clarifying the wording of the directive in this respect. The conditions set out in the CEMFAW Regulations (for both the main MRI exemption and the discretionary exemption) are as follows:

(i) the exposure of employees to EMFs is as low as is reasonably practicable and
(ii) employees are protected against any health effects and safety risks related to that exposure.

Senior staff of MRI facilities will be familiar with the steps necessary to ensure the health and safety of staff (as well as patients and visitors) and so comply with condition (ii). These will include local rules and procedures setting out safe working practices, appropriate staff training, incorporation of safety considerations into the design of the MRI unit and control of access to the MRI suite including robust screening procedures and labelling of equipment. Very sound advice on all of these aspects is contained in the Medicines and Healthcare Products Regulatory Agency (MHRA) guidance on the safe use of MRI equipment,^10^ which is referred to specifically in the HSE guide to the regulations.^8^ The MHRA guidance includes recommendations on the appointment of appropriate dutyholders, primarily the MR Responsible Person and the MR Safety Expert. It is also important to remember that an MRI scanner that is CE-marking under the Medical Devices Directive is designed to be safe if used in accordance with the manufacturer instructions for use.

Condition (i) requires a careful interpretation and understanding of the context. The terminology “as low as reasonably practicable” (ALARP) is familiar to the imaging community in the context of ionizing radiation. In that instance, there is assumed to be a stochastic risk from any exposure right down to zero dose. However, known physiological and health effects of EMF occur only above certain threshold levels of exposure (which are considerably higher than the ELVs, as these incorporate wide safety margins), and so application of ALARP to MRI is different. It is clear that when exposure is below the ELVs, no action is needed. For higher exposures, the HSE guide states that “you must ensure the ELVs are exceeded to the lowest extent reasonably practicable”. What is “reasonable” in this instance clearly depends on the nature and magnitude of the risk that is being mitigated. For example, although staff working close to the bore of an MRI scanner may exceed the ELVs relating to exposure to the switched gradients, physiological effects usually arise only for people actually inside the bore, when the considerably higher threshold for peripheral nerve stimulation may be exceeded for some particularly sensitive individuals. Given this knowledge, it would clearly be inappropriate and disproportionate to restrict routine clinical activity close to the bore simply in order to reduce exposure, or to prohibit the presence of staff (or patient escorts) in the scanner room during imaging. It would be sensible, however, to train staff not to lean into the bore itself during imaging unless this is clinically justified. Interpreted in this way, the ALARP requirement translates into an issue of training staff to avoid situations in which physiological effects may arise, rather than avoidance of exposure on a purely precautionary basis.

The exemption means that a number of provisions of the regulations, such as the need to produce an action plan to ensure compliance with the ELVs, do not apply to MRI facilities. However, it is important to realize that the regulations are not just about exposure limits, and other requirements do still apply. Fortunately, these mostly relate to measures that should already be in place in MRI facilities. There is a requirement to *assess* levels of EMF exposure: importantly, *not* to measure or calculate actual exposure levels. This assessment can be based on manufacturer data and guidance from a variety of sources, including professional bodies such as the BIR. It could simply consist of recognizing, on the basis of published literature,^11,12^ that working close to MRI scanners can result in the ELVs being exceeded. Because of the MRI exemption, it is not necessary to show that the ELVs actually are exceeded in any specific case, or to take any action as long as the conditions attached to the exemption are satisfied. Having assessed EMF exposure, it is then necessary to undertake a risk assessment. The HSE guide^8^ gives good advice on what to consider in this process. Risk assessments are already a requirement of existing health and safety legislation and so should already be in place for MRI units. They may, however, need to be reviewed to ensure that they meet the requirements of the regulations, *e.g.* that they address EMF-related risks explicitly. This is a “one off” task that should be undertaken by the MR Responsible Person, supported by the MR Safety Expert.^10^ Where a facility has multiple MR systems, a single set of risk assessments will normally be sufficient, unless the nature of the work undertaken with specific systems results in additional risks. The BIR has developed a set of generic risk assessments, also addressing the requirement to assess EMF exposure, which we hope will be of use to MRI facilities in this regard. They are available from http://www.bir.org.uk/professional-resources/special-interest-groups/bir-magnetic-resonance/emf-risk-assessments/. It is also necessary to provide staff with relevant information and training and to make health surveillance available if a member of staff reports experiencing a health effect owing to EMF exposure (something that will already be available through occupational health services in this very unlikely eventuality). Training will already be in place for staff in MRI facilities, but again there may be a need for the MR Responsible Person and MR Safety Expert to review existing provision and ensure that it addresses the issues recommended by the HSE^8^ and MHRA.^10^

We believe that by adopting the CEMFAW Regulations, the UK has met its obligation to transpose the directive in a way that is sensible and proportionate. The regulations should have minimal effect on the operation of MRI facilities and create minimal additional work for MRI staff. Given the situation a few years ago, and the potential for disruption of clinical and research MRI, this is a remarkable achievement.

## References

[1] The Control of Electromagnetic Fields at Work Regulations 2016, SI 2016/588. [Accessed 2 September 2016] Available from: http://www.legislation.gov.uk/uksi/2016/588/pdfs/uksi_20160588_en.pdf

[2] The Control of Electromagnetic Fields at Work Regulations (Northern Ireland) 2016, SR 2016/266. [Accessed 2 September 2016]. Available from: http://www.legislation.gov.uk/nisr/2016/266/pdfs/nisr_20160266_en.pdf

[3] Directive 2013/35/EU of the European Parliament and of the Council. [Accessed 2nd September 2016]. Available from: http://new.eur-lex.europa.eu/legal-content/EN/TXT/PDF/?uri=CELEX:32013L0035&qid=1373881688213&from=EN

[4] Directive 2004/40/EC of the European Parliament and of the Council. [Accessed 2 September 2013]. Available from: http://eur-lex.europa.eu/legal-content/EN/TXT/PDF/?uri=CELEX:32004L0040&from=EN

[5] Keevil SF, Gedroyc W, Gowland P, Hill DL, Leach MO, Ludman CN (2005). Electromagnetic field exposure limitation and the future of MRI. *Br J Radiol*.

[6] Keevil SF, Krestin GP (2010). EMF directive still poses a risk to MRI research in Europe. *Lancet*.

[7] Keevil SF, Lomas DJ (2013). The European union physical agents (electromagnetic fields) directive: an update for the MRI community. *Br J Radiol*.

[8] Health and Safety Executive. Electromagnetic fields at work. A guide to the control of electromagnetic fields at Work Regulations 2016. London: HSE; 2016. [Accessed 5 September 2016]. Available from: http://www.hse.gov.uk/pubns/priced/hsg281.pdf

[9] European Commission. Non-binding guide to good practice for implementing Directive 2013/35/EU electromagnetic fields. Vol. 1: Practical Guide. Luxembourg: Publications Office of the European Union; 2015. [Accessed 5 September 2016]. Available from: http://ec.europa.eu/social/main.jsp?catId=738&langId=en&pubId=7845&type=2&furtherPubs=yes

[10] Medicines and Healthcare Products Regulatory Agency. Safety guidelines for magnetic resonance imaging equipment in clinical use, v. 4.2. London: MHRA; 2015. [Accessed 2 September 2016]. Available from: http://www.gov.uk/government/uploads/system/uploads/attachment_data/file/476931/MRI_guidance_2015_-_4-02d1.pdf

[11] Crozier S, Wang H, Trakic A, Liu F (2007). Exposure of workers to pulsed gradients in MRI. *J Magn Reson Imaging*.

[12] Crozier S, Trakic A, Wang H, Liu F (2007). Numerical study of currents in workers induced by body-motion around high-ultrahigh field MRI magnets. *J Magn Reson Imaging*.

